# Recurrent Aggressive Angiomyxoma That Responded to the Gonadotropin-Releasing Hormone (GnRH) Antagonist Relugolix

**DOI:** 10.7759/cureus.81270

**Published:** 2025-03-27

**Authors:** Arisa Saito, Shinichi Komiyama, Masaru Nagashima, Tsuyoki Kugimiya, Takahumi Mukai

**Affiliations:** 1 Department of Obstetrics and Gynecology, Toho University Omori Medical Center, Tokyo, JPN

**Keywords:** aggressive angiomyxoma, gnrh antagonist, hormone therapy, recurrence, relugolix

## Abstract

Aggressive angiomyxoma (AAM) is a rare benign mesenchymal tumor occurring in the vulva, vagina, and pelvis; however, it is difficult to treat because it often recurs locally. Since AAM expresses the estrogen receptor (ER) and progesterone receptor (PgR), various hormone therapies have been reportedly effective. Regardless, the efficacy of gonadotropin-releasing hormone (GnRH) antagonists has not yet been reported. We encountered a case of relapsed AAM in which the GnRH antagonist relugolix was highly effective. Although the patient initially underwent surgical resection for the AAM, recurrence occurred 11 months postoperatively. Since the immunohistochemical expression of ER and PgR was positive, relugolix (40 mg/day) was orally administered daily for six months, resulting in significant tumor regression (8.7 cm to 4.6 cm). This is the first report of successful GnRH antagonist treatment for AAM. Although AAM may be difficult to treat, GnRH antagonists are promising candidates for hormone therapy.

## Introduction

Aggressive angiomyxoma (AAM) is a rare benign mesenchymal tumor commonly occurring in the perineum, vulva, vagina, and pelvis [1]. Histopathologically, it is a benign tumor that often recurs locally and, hence, is difficult to treat [2]. Conversely, since AAM expresses estrogen receptors (ERs) and progesterone receptors (PgRs) at a high rate, hormone therapy with gonadotropin-releasing hormone (GnRH) agonists and aromatase inhibitors has been performed with adequate efficacy [3]. However, GnRH antagonist use has not yet been reported. Here, we report a case in which relugolix, a GnRH antagonist, was substantially effective for the postoperative recurrence of AAM.

## Case presentation

**Figure 1:**
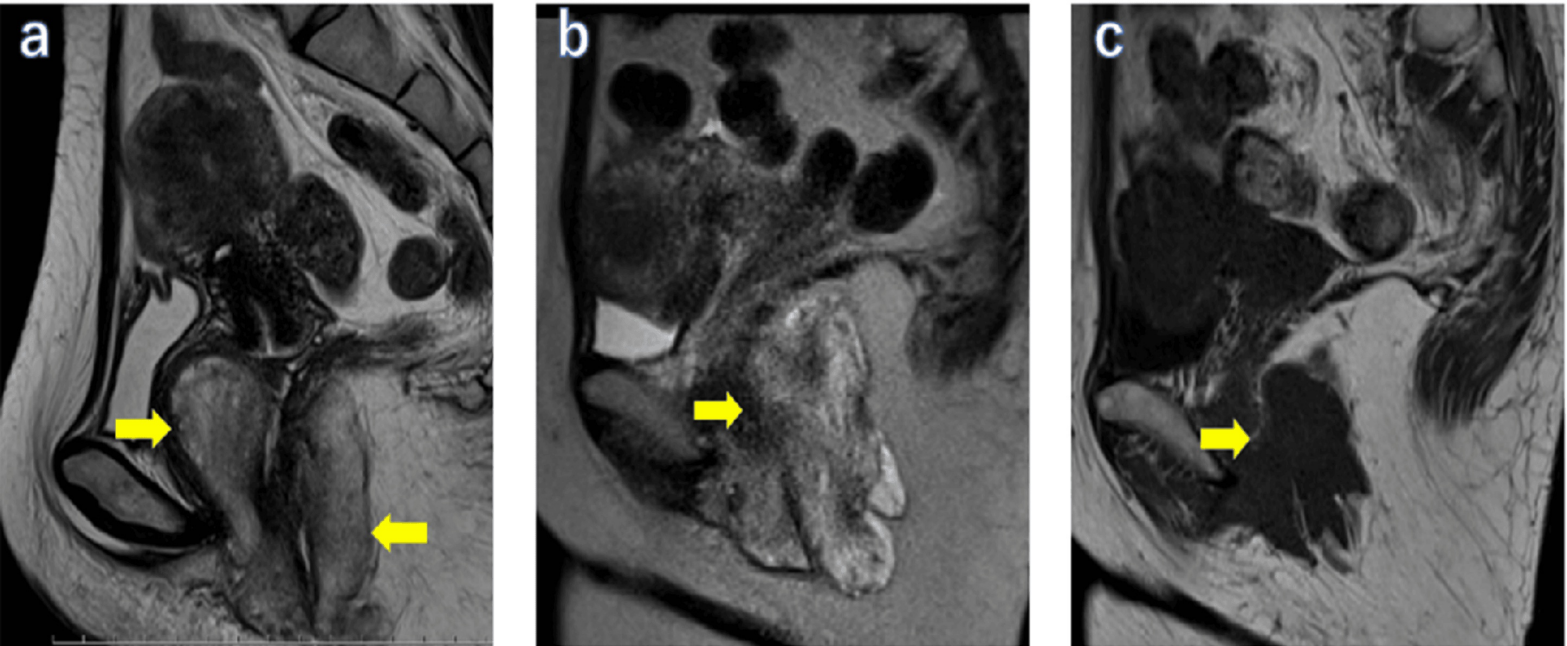
T2-weighted sagittal image of the patient's pelvic cavity MRI MRI: magnetic resonance imaging (a) Initial visit, (b) recurrence (11 months postoperatively), (c) the end of GnRH antagonist treatment (18 months postoperatively). A lobulated tumor with a diameter of 8 cm is seen from the left side of the vulva to the subvaginal mucosa, and the inside of the tumor shows a swirling and layering pattern (arrow) (a). A mass with a diameter of 8.6 cm and severe edematous changes similar to those seen at the initial examination are shown (b). Significantly reduced tumor of 4.6 cm after six months of relugolix administration (c)

The patient was a 44-year-old woman (height: 165 cm; weight: 64 kg) with a regular menstrual cycle of 29 days. Past medical history included a bilateral inguinal hernia. There was no medication that she took regularly. She noticed vulvar discomfort 18 months ago, which gradually worsened. She visited a local clinic with a chief complaint of vulvar swelling and was referred to our department (Toho University Omori Medical Center, Ota-ku, Tokyo, Japan) for a detailed examination. Vaginal examination revealed a mass bulging from the left side of the vaginal wall, filling the vagina, and transvaginal sonography revealed a solid tumor. Magnetic resonance imaging (MRI) of the pelvic cavity revealed a lobulated mass with a long axis of 8 cm extending from the left side of the vulva to the subvaginal mucosa. T2-weighted images showed a swirling and layering pattern inside the tumor, suggesting AAM (Figure 1a). Angiomyxoma was suspected on a transvaginal tumor biopsy; therefore, we decided to remove it surgically.

After the transvaginal incision of the mucosa and digital examination of the rectum, the boundary between the tumor and the rectal wall was dissected, releasing the tumor. The distinct border between the normal tissue and the tumor made the peeling process easy. The deep extension of the cranial margin of the tumor into the pelvic cavity renders it challenging to be identified by transvaginal manipulation; therefore, we performed a transabdominal laparotomy with left rectal cavity extension. After identifying the cranial margin of the tumor under direct view, the entire tumor was removed through a transabdominal approach. No gross residual tumor was found in the pelvic cavity after resection.

**Figure 2:**
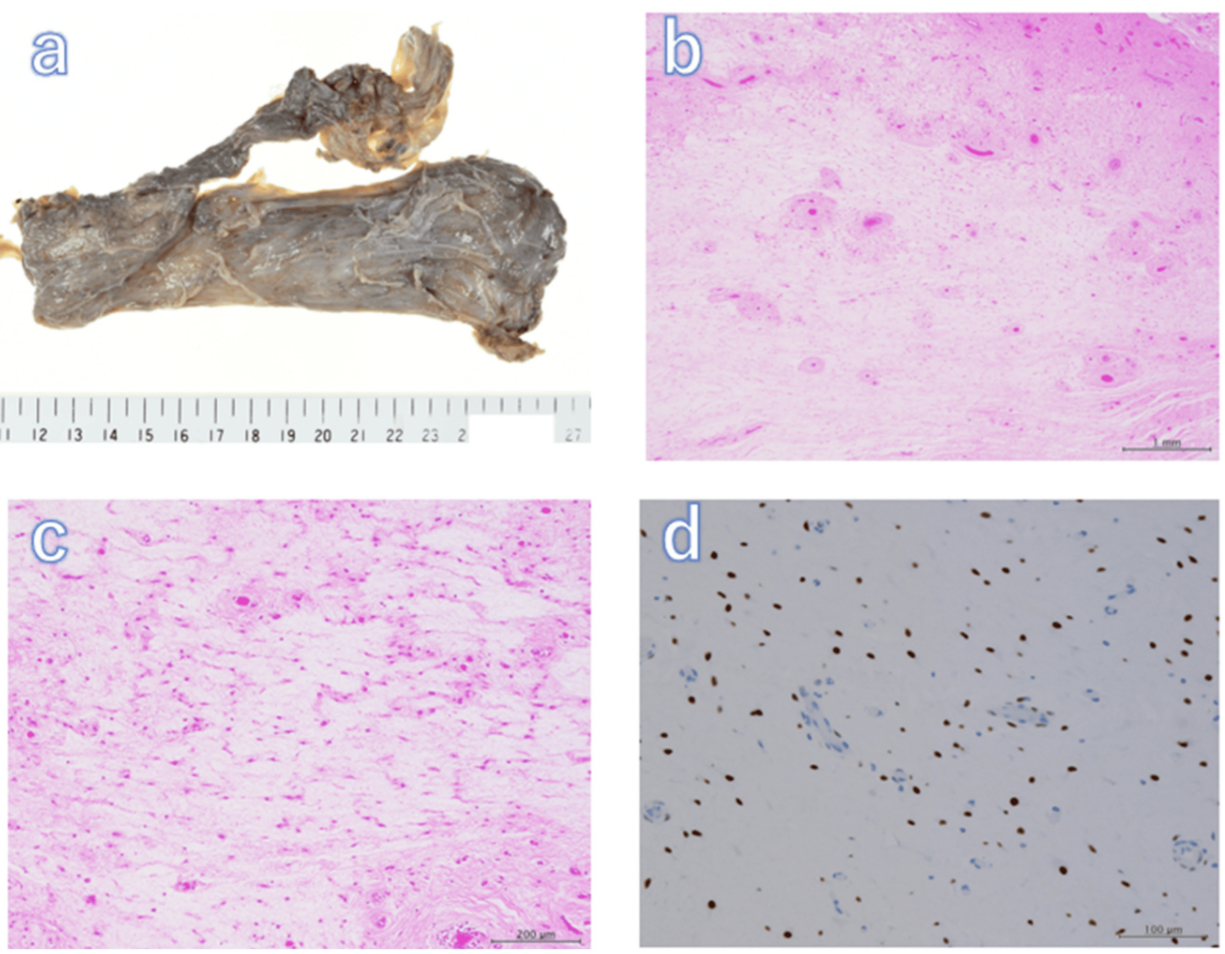
Pathological findings of the tumor (a) Macroscopic image. (b) Hematoxylin-eosin (HE)-stained low magnification image (2x objective). (c) HE staining at high magnification (10x objective). (d) Immunohistochemistry of estrogen receptor (ER) stained image. Macroscopically, the tumor is soft and elastic (a). Histologically, a myxedematous interstitium is shown in the background, with sparse proliferation of spindle-shaped cells with minimal atypia. Conspicuous interposition of small to medium-sized blood vessels (b, c). ER expression in the nucleus of tumor cells (d)

Histopathological examination of the resected specimen revealed that the tumor had a myxedematous interstitial background with sparse proliferation of spindle-shaped cells and no atypia. The interposition of small-to-medium-sized blood vessels was conspicuous, and some blood vessels had thickened walls and showed hyalinization. In addition, the proliferation of collagen fibers was observed mainly at the tumor margins. Immunohistochemistry was positive for ER, PgR, desmin, and smooth muscle actin (SMA) (Figure 2). Based on these findings, the pathological diagnosis of AAM was confirmed.

The postoperative course was uneventful, and the patient was discharged on the fifth postoperative day. Subsequently, she underwent regular outpatient surveillance, and a pelvic examination 11 months postsurgery revealed suspicious findings for tumor recurrence. MRI revealed a solid tumor approximately 8 cm in length extending from the left side of the vagina to the subcutaneous area of the left vulva, diagnosed as an AAM recurrence (Figure 1b).

Considering the following factors, the patient strongly desired treatment other than surgery: The tumor tissue expressed ER and PgR, she was not menopausal and had preserved ovarian function; hence, further treatment was aimed at suppressing estrogen and progesterone. Hormone therapy was initiated. With the patient’s informed consent, we decided to perform daily oral administration of the GnRH antagonist, relugolix (40 mg/day), for six months. After starting relugolix, the tumor gradually shrank, and after six months of continued oral administration, it significantly shrank from 8.7 cm to 4.6 cm (partial response) (Figure 1c). No adverse events were noted concerning relugolix except a mild hot flush, and the patient could complete the oral administration course without dose reduction or discontinuation. At the end of relugolix treatment, the patient was followed up, and eight months after the recurrence diagnosis, the tumor remained the same size with no signs of relapse. Although she is currently under observation, if the tumor grows again, we would consider readministration of relugolix. A written informed consent was obtained from the patient for reporting this case.

## Discussion

AAM is a clinicopathological concept proposed by Steeper and Rosa in 1983 and is a mesenchymal tumor that commonly occurs in the perineum, vulva, vagina, and pelvis [1]. It is overwhelmingly more frequent in women, with a male-to-female ratio of 1:6.6, and the general age of onset is reportedly between 20 and 40 [4]. Though a benign tumor histopathologically, it shows signs of angiogenesis and invasion and is characterized by frequent local recurrence [2]. The abundant blood flow inside a tumor increases rapidly and may bleed profusely during scraping or biopsy; therefore, careful clinical handling is necessary [5].

MRI of AAM shows a low signal on T1-weighted images and a high signal on T2-weighted images, with a discernible tumor border. It is characterized by a “swirling and layering pattern,” in which structures, mainly cordlike or layered blood vessels, exist in a spiral shape inside the tumor [6].

The pathological findings were characterized by the proliferation of large and small blood vessels and small spindle-shaped cells with minimal atypia and inconspicuous nuclear division in the myxedematous interstitium. The tumor is unencapsulated and may invade muscles, nerves, and fatty tissues. Immunohistochemical staining often yields positive results for vimentin, desmin, ER, and PgR [7].

Surgical resection is the basis of treatment, and complete resection should be performed. However, the recurrence rate after resection is reported to be 9%-72%, which does not change, even with residual disease after gross complete resection [4]. Surgery alone cannot achieve a complete cure, which may be because the tumor was unencapsulated and spread microscopically beyond its macroscopic boundaries.

Positive immunohistochemical staining for ER and PgR and the age of onset suggest a relationship with sex hormones, and hormone therapy can reduce tumor size [3]. To date, 18 patients in the scientific literature have reported successful treatment of recurrent AAM with hormone therapy [8-13], as summarized in Table 1. Although most patients respond to hormone therapy, the duration of response is short and may indicate repeated hormone therapy in young patients. Since the probability of patient relapse is high, we will need to search for further treatments in the future.

**Table 1:** Summary of scientific literature on effective hormone therapy cases for recurrent aggressive angiomyxoma AI; aromatase inhibitor; CR: complete response; ER: estrogen receptor; GnRH: gonadotropin-releasing hormone; Mo: month; NA: not available; PgR: progesterone receptor; PR: partial response; Ref: references; SD: stable disease; SERM: selective estrogen receptor modulator

No	Age	Tumor site	ER	PgR	Type of hormone therapy	Best response	Time to responcse（mo)	Ref
1	27	Vulva	Positive	Positive	GnRH agonist	CR	3	[9]
2	34	Perineum	Positive	Positive	GnRH agonist	CR	3	[13]
3	36	Rectovaginal septum	Positive	Positive	GnRH agonist	CR	6	[11]
4	46	Perineum	Positive	Positive	GnRH agonist	CR	5	[12]
5	36	Pelvis	Positive	Positive	GnRH agonist	PR	2	[8]
6	48	Pelvis	Positive	Positive	GnRH agonist	CR	2	[10]
7	20	Pelvis	Positive	Positive	GnRH agonist	PR	4	[10]
8	36	Pelvis	NA	NA	GnRH agonist	SD	3	[10]
9	47	Pelvis	Positive	NA	GnRH agonist	SD	NA	[10]
10	53	Pelvis	Positive	Positive	GnRH agonist	SD	3	[10]
11	61	Pelvis	Positive	Positive	AI	SD	NA	[10]
12	63	Pelvis	Positive	Positive	AI	SD	1	[10]
13	40	Perineum	Positive	Positive	SERM	PR	NA	[10]
14	35	Pelvis	Positive	Positive	SERM	PR	3	[10]
15	45	Pelvis	Positive	Positive	GnRH agonist + SERM	CR	NA	[10]
16	43	Perineum	Positive	Positive	GnRH agonist + SERM	PR	NA	[10]
17	46	Perineum	Positive	Positive	Tamoxifen→GnRH agonist	PR	3	[10]
18	42	Perineum	Positive	Positive	GnRH agonist	SD	NA	[10]
our case	44	Pelvis	Positive	Positive	GnRH antagonist	PR	8	―

Relugolix is the first orally administered GnRH antagonist. Unlike existing drugs, it has a selective antagonistic effect on the GnRH receptor and inhibits the secretion of luteinizing and follicle-stimulating hormones, strongly suppressing estrogen and progesterone. The hospital visits can be markedly reduced with orally administered GnRH antagonist preparations compared with injectable GnRH agonist preparations. Furthermore, major advantages include the absence of injection pain and estrogen flare-up phenomenon, which are characteristics of GnRH agonists [14]. Although many reported on the use of GnRH agonists as a hormone therapy for AAM, to our knowledge, GnRH antagonist usage has not been reported. This is the first report demonstrating the effectiveness of GnRH antagonists against AAM. In addition to relugolix, other GnRH antagonists include elagolix and linzagolix, which are also expected to be effective therapies for AAM. In the future, we believe that GnRH antagonists will prove beneficial as hormonal therapies for AAM and we need further studies for it. Problems encountered while selecting a GnRH antagonist include the poor compliance issue affecting its efficacy, which may increase as it is an oral drug, and its effect on bone metabolism in premenopausal women. Therefore, long-term drug administration should be avoided [15]. Furthermore, it may be necessary to consider sequential or rotational administration of other hormonal drugs (such as progestins, aromatase inhibitors, and selective estrogen receptor modulators (SERMs)) to prevent relapse after hormone therapy completion.

In conclusion, to our knowledge, this is the first report of a patient with AAM who responded significantly to a GnRH antagonist. Although AAM can be difficult to treat after recurrence, GnRH antagonists hold great promise as hormone therapy for these patients.

## Conclusions

To our knowledge, this is the first report of a patient with AAM who responded significantly to a GnRH antagonist. Although AAM can be difficult to treat after recurrence, GnRH antagonists hold great promise as hormone therapy for these patients.
